# Supplementary material for: A Likelihood Approach for Real-Time Calibration of Stochastic Compartmental Epidemic Models
Source: PLoS Comput Biol. 2017 Jan 17;13(1):e1005257. doi: 10.1371/journal.pcbi.1005257 (PMC5240920; doi:10.1371/journal.pcbi.1005257)
Supplement: S1 File — (TAR.GZ) [file pcbi.1005257.s014.tar.gz › HSPH_Online-SI-Revision/IPoi09/n100-extreme/n100-extreme_BENCH_table8.pdf]

1	R0	{2.76052, 16.3913}	{2.8939, 16.8013}	{{2.8939, 16.8013}, {2.8939, 16.8013}}	{4.8317, 2.50133}	inf	3200.	5900.92	{2957., 10799.}	88.3832	0.223355	newly	1155.	2120.26	{1032., 3850.}	88.103	0.221576	Reff	0.245824	0.246176	{0.217592, 0.29266}	11.3085	ar	92090.	93276.2	{93112., 93387.}	1.28812
2	R0	{2.18202, 15.1734}	{2.76795, 19.6041}	{{2.76795, 19.6041}, {2.76795, 19.6041}}	{26.8526, 29.2004}	inf	3327.	3768.18	{1785., 5942.}	50.9443	0.186987	newly	1337.	1182.23	{539., 1929.}	48.6396	0.229652	Reff	0.385257	0.248854	{0.226944, 0.270789}	35.4059	ar	84264.	92211.6	{92022., 92408.}	9.43177
3	R0	{2.57891, 12.5971}	{2.99911, 15.6207}	{{2.99911, 15.6207}, {2.99911, 15.6207}}	{16.2937, 24.0023}	inf	1362.	2622.79	{1214., 4737.}	97.8175	0.235424	newly	609.	988.839	{463., 1749.}	73.7804	0.194061	Reff	0.262765	0.198316	{0.185345, 0.212997}	24.7167	ar	90302.	94013.6	{93846., 94150.}	4.11026
4	R0	{2.5856, 9.28508}	{2.93653, 10.872}	{{2.93653, 10.872}, {2.93653, 10.872}}	{13.5725, 17.0911}	inf	433.	1090.3	{537., 2022.}	151.861	0.332371	newly	254.	549.803	{265., 1007.}	116.755	0.267778	Reff	0.254526	0.196161	{0.189494, 0.203678}	22.9311	ar	90312.	93586.3	{93426., 93765.}	3.62556
5	R0	{2.4406, 12.1341}	{2.99911, 15.6207}	{{2.99911, 15.6207}, {2.99911, 15.6207}}	{22.8841, 28.7339}	inf	1891.	3723.53	{1737., 6669.}	101.282	0.243054	newly	894.	1408.15	{643., 2519.}	70.5835	0.189922	Reff	0.297534	0.207536	{0.189934, 0.228352}	30.6338	ar	88597.	94013.6	{93846., 94150.}	6.1138
6	R0	{2.72155, 15.4094}	{2.8939, 16.8013}	{{2.8939, 16.8013}, {2.8939, 16.8013}}	{6.33279, 9.0328}	inf	3044.	3161.07	{1547., 5860.}	46.0433	0.18427	newly	1132.	1127.92	{565., 2167.}	46.9487	0.196206	Reff	0.252533	0.219346	{0.204483, 0.241959}	14.5656	ar	91682.	93276.2	{93112., 93387.}	1.73887
7	R0	{2.63781, 14.3935}	{2.96978, 18.8857}	{{2.96978, 18.8857}, {2.96978, 18.8857}}	{12.5851, 31.2099}	inf	2374.	6683.52	{3070., 11944.}	181.53	0.377395	newly	968.	2168.58	{1013., 3905.}	125.327	0.280817	Reff	0.252043	0.24369	{0.204974, 0.291395}	17.4969	ar	91192.	93825.4	{93652., 93970.}	2.88776
8	R0	{2.82391, 16.104}	{2.8939, 16.8013}	{{2.8939, 16.8013}, {2.8939, 16.8013}}	{2.47848, 4.32998}	inf	3239.	4331.61	{2172., 7999.}	53.574	0.164644	newly	1178.	1546.7	{763., 2841.}	53.0699	0.165763	Reff	0.232408	0.230232	{0.210444, 0.262274}	8.52531	ar	92664.	93276.2	{93112., 93387.}	0.660698
9	R0	{2.80645, 14.6457}	{2.96398, 17.7348}	{{2.96398, 17.7348}, {2.96398, 17.7348}}	{5.61314, 21.0922}	inf	2875.	7160.74	{3583., 12914.}	149.069	0.337736	newly	1160.	2467.16	{1219., 4488.}	113.498	0.269237	Reff	0.229399	0.251046	{0.208812, 0.30855}	14.7449	ar	92567.	93780.3	{93638., 93879.}	1.31068
10	R0	{1.86213, 5.4271}	{2.93653, 10.872}	{{2.93653, 10.872}, {2.93653, 10.872}}	{57.6974, 100.328}	inf	95.	649.492	{333., 1268.}	583.676	0.763694	newly	92.	323.574	{167., 653.}	251.711	0.475293	Reff	0.460598	0.19296	{0.186851, 0.200653}	58.1067	ar	75362.	93586.3	{93426., 93765.}	24.1824
11	R0	{2.27349, 14.6338}	{2.96978, 18.8857}	{{2.96978, 18.8857}, {2.96978, 18.8857}}	{30.6265, 29.0553}	inf	2757.	5112.29	{2280., 9108.}	92.0578	0.221878	newly	1137.	1649.55	{724., 2931.}	63.3699	0.177469	Reff	0.351982	0.225949	{0.198025, 0.259648}	37.0688	ar	85805.	93825.4	{93652., 93970.}	9.34724
12	R0	{2.77261, 14.1894}	{2.8833, 17.1847}	{{2.8833, 17.1847}, {2.8833, 17.1847}}	{3.99227, 21.1094}	inf	2393.	5252.26	{2516., 9575.}	120.908	0.280411	newly	959.	1849.28	{886., 3385.}	97.4359	0.236814	Reff	0.226855	0.241984	{0.215469, 0.277979}	10.1322	ar	92508.	93197.4	{93009., 93386.}	0.745208
13	R0	{2.65402, 19.3935}	{2.93978, 19.9817}	{{2.93978, 19.9817}, {2.93978, 19.9817}}	{10.7671, 3.03297}	inf	6091.	4625.54	{2275., 7883.}	50.7562	0.266875	newly	2008.	1414.28	{694., 2424.}	53.3889	0.295824	Reff	0.292659	0.225451	{0.202639, 0.256143}	25.8139	ar	91184.	93627.3	{93451., 93834.}	2.6795
14	R0	{1.75297, 4.45354}	{2.86001, 13.5542}	{{2.86001, 13.5542}, {2.86001, 13.5542}}	{63.1523, 204.347}	inf	33.	2608.87	{1157., 3990.}	7805.66	1.82046	newly	37.	1117.46	{495., 1703.}	2920.16	1.40079	Reff	0.50468	0.22097	{0.206464, 0.233892}	56.2157	ar	71263.	92998.7	{92780., 93186.}	30.5006
15	R0	{1.99928, 7.1501}	{2.99939, 13.7076}	{{2.99939, 13.7076}, {2.99939, 13.7076}}	{50.0235, 91.712}	inf	273.	1806.74	{942., 2965.}	561.81	0.759364	newly	197.	754.726	{390., 1262.}	283.11	0.521271	Reff	0.411872	0.19148	{0.182963, 0.199999}	53.5099	ar	79602.	94036.2	{93909., 94200.}	18.133
16	R0	{2.67398, 10.3134}	{2.86001, 13.5542}	{{2.86001, 13.5542}, {2.86001, 13.5542}}	{6.95705, 31.4232}	inf	624.	1748.84	{766., 2684.}	180.262	0.36798	newly	342.	739.279	{324., 1139.}	119.701	0.27016	Reff	0.237957	0.213608	{0.20329, 0.222423}	11.0218	ar	91289.	92998.7	{92780., 93186.}	1.87281
17	R0	{2.44634, 16.3226}	{2.93978, 19.9817}	{{2.93978, 19.9817}, {2.93978, 19.9817}}	{20.1705, 22.4174}	inf	3997.	7631.56	{3968., 13084.}	93.3561	0.230405	newly	1461.	2371.75	{1221., 4091.}	71.2604	0.188114	Reff	0.317486	0.260923	{0.216603, 0.317114}	28.0237	ar	88710.	93627.3	{93451., 93834.}	5.54309
18	R0	{2.02168, 10.1676}	{2.97157, 19.3547}	{{2.97157, 19.3547}, {2.97157, 19.3547}}	{46.9852, 90.3566}	inf	1489.	7597.74	{3537., 13056.}	410.258	0.64212	newly	847.	2425.32	{1130., 4177.}	186.343	0.387276	Reff	0.420772	0.254465	{0.210684, 0.305804}	42.7133	ar	80299.	93849.1	{93703., 93993.}	16.8745
19	R0	{2.89969, 15.009}	{2.8939, 16.8013}	{{2.8939, 16.8013}, {2.8939, 16.8013}}	{0.199677, 11.9415}	inf	2686.	5900.92	{2957., 10799.}	120.596	0.284337	newly	1026.	2120.26	{1032., 3850.}	108.718	0.260626	Reff	0.211938	0.246176	{0.217592, 0.29266}	16.3544	ar	93349.	93276.2	{93112., 93387.}	0.105738
20	R0	{2.68933, 7.34928}	{2.93653, 10.872}	{{2.93653, 10.872}, {2.93653, 10.872}}	{9.19188, 47.9329}	inf	136.	1090.3	{537., 2022.}	701.688	0.835055	newly	80.	549.803	{265., 1007.}	587.254	0.768199	Reff	0.227975	0.196161	{0.189494, 0.203678}	13.955	ar	91574.	93586.3	{93426., 93765.}	2.19747
21	R0	{2.15352, 9.28074}	{2.86001, 13.5542}	{{2.86001, 13.5542}, {2.86001, 13.5542}}	{32.8063, 46.0465}	inf	838.	2608.87	{1157., 3990.}	211.321	0.415726	newly	504.	1117.46	{495., 1703.}	124.398	0.279343	Reff	0.358733	0.22097	{0.206464, 0.233892}	38.4026	ar	83845.	92998.7	{92780., 93186.}	10.9174
22	R0	{2.21953, 9.04387}	{2.79531, 12.1249}	{{2.79531, 12.1249}, {2.79531, 12.1249}}	{25.9415, 34.0676}	inf	694.	1701.87	{881., 2454.}	145.879	0.328934	newly	414.	792.583	{416., 1161.}	95.3824	0.237562	Reff	0.343938	0.224312	{0.214875, 0.23243}	34.7813	ar	84856.	92457.4	{92317., 92603.}	8.95794
23	R0	{2.21987, 14.5347}	{2.76795, 19.6041}	{{2.76795, 19.6041}, {2.76795, 19.6041}}	{24.6897, 34.8779}	inf	2885.	6244.98	{3061., 9888.}	117.809	0.276129	newly	1192.	1972.13	{927., 3181.}	74.9587	0.198075	Reff	0.366057	0.277549	{0.238265, 0.322272}	28.4352	ar	85067.	92211.6	{92022., 92408.}	8.39878
24	R0	{2.84268, 17.9659}	{2.78106, 19.8054}	{{2.76795, 19.6041}, {2.79269, 19.984}}	{2.1676, 10.2391}	inf	4541.	6365.53	{2704., 12191.}	63.3747	0.189528	newly	1477.	2022.8	{840., 3749.}	63.3199	0.190897	Reff	0.242139	0.297191	{0.23439, 0.33733}	24.0468	ar	92836.	92317.6	{92084., 92517.}	0.558377
25	R0	{2.52706, 10.3679}	{2.8833, 17.1847}	{{2.8833, 17.1847}, {2.8833, 17.1847}}	{14.097, 65.7491}	inf	746.	5252.26	{2516., 9575.}	604.057	0.779881	newly	432.	1849.28	{886., 3385.}	328.074	0.560665	Reff	0.269107	0.241984	{0.215469, 0.277979}	16.7108	ar	89623.	93197.4	{93009., 93386.}	3.98824
26	R0	{2.62835, 19.1088}	{2.93978, 19.9817}	{{2.93978, 19.9817}, {2.93978, 19.9817}}	{11.8489, 4.56805}	inf	5501.	5968.61	{3017., 10190.}	45.8617	0.173015	newly	1758.	1840.89	{920., 3184.}	46.2653	0.180802	Reff	0.292378	0.240086	{0.209636, 0.281925}	24.5945	ar	90908.	93627.3	{93451., 93834.}	2.99124
27	R0	{2.76831, 19.4155}	{2.96978, 18.8857}	{{2.96978, 18.8857}, {2.96978, 18.8857}}	{7.27773, 2.72875}	inf	4502.	3874.84	{1741., 6947.}	51.6831	0.247933	newly	1418.	1249.37	{549., 2262.}	51.8802	0.243777	Reff	0.252857	0.213711	{0.19357, 0.236781}	18.7949	ar	92175.	93825.4	{93652., 93970.}	1.79051
28	R0	{2.96609, 16.8903}	{2.95209, 14.037}	{{2.95209, 14.037}, {2.95209, 14.037}}	{0.472002, 16.8931}	inf	3132.	1690.47	{898., 3272.}	56.2976	0.36797	newly	1123.	694.145	{368., 1348.}	53.3062	0.321616	Reff	0.198639	0.198081	{0.190616, 0.209274}	3.3263	ar	94024.	93710.9	{93555., 93857.}	0.332997
29	R0	{2.17046, 14.39}	{2.76795, 19.6041}	{{2.76795, 19.6041}, {2.76795, 19.6041}}	{27.5283, 36.2342}	inf	3337.	7919.1	{3988., 12551.}	137.476	0.317528	newly	1397.	2519.92	{1223., 3964.}	85.4102	0.218197	Reff	0.390705	0.30096	{0.245711, 0.358283}	30.0086	ar	84001.	92211.6	{92022., 92408.}	9.7744
30	R0	{2.88772, 12.4647}	{2.93653, 10.872}	{{2.93653, 10.872}, {2.93653, 10.872}}	{1.69026, 12.7777}	inf	1402.	649.492	{333., 1268.}	59.8384	0.426053	newly	651.	323.574	{167., 653.}	57.9764	0.399806	Reff	0.203498	0.19296	{0.186851, 0.200653}	5.31755	ar	93273.	93586.3	{93426., 93765.}	0.339142
31	R0	{2.46831, 8.14308}	{2.98388, 9.60029}	{{2.98388, 9.60029}, {2.98388, 9.60029}}	{20.8876, 17.8951}	inf	300.	478.403	{256., 700.}	66.7688	0.175391	newly	189.	261.129	{139., 396.}	53.7634	0.155296	Reff	0.280894	0.183832	{0.178347, 0.190819}	34.5546	ar	88762.	93948.	{93795., 94112.}	5.84257
32	R0	{1.93236, 7.38987}	{2.9884, 12.8326}	{{2.9884, 12.8326}, {2.9884, 12.8326}}	{54.6503, 73.6512}	inf	389.	1572.	{818., 2500.}	304.113	0.543751	newly	288.	690.968	{350., 1087.}	140.334	0.318536	Reff	0.437467	0.190691	{0.184414, 0.197533}	56.4102	ar	77654.	93983.5	{93851., 94123.}	21.0286
33	R0	{2.82251, 17.6954}	{2.96947, 15.963}	{{2.96947, 15.963}, {2.96947, 15.963}}	{5.20671, 9.79011}	inf	4866.	3313.9	{1548., 6014.}	54.7446	0.306098	newly	1670.	1228.21	{565., 2281.}	53.3658	0.28192	Reff	0.244147	0.209106	{0.1926, 0.236192}	17.3628	ar	92745.	93829.	{93704., 93974.}	1.16878
34	R0	{1.87083, 12.3994}	{2.76795, 19.6041}	{{2.76795, 19.6041}, {2.76795, 19.6041}}	{47.953, 58.1052}	inf	3095.	6244.98	{3061., 9888.}	104.338	0.25148	newly	1528.	1972.13	{927., 3181.}	53.8645	0.174171	Reff	0.498595	0.277549	{0.238265, 0.322272}	44.6647	ar	76282.	92211.6	{92022., 92408.}	20.8825
35	R0	{2.95779, 16.3003}	{2.96947, 15.963}	{{2.96947, 15.963}, {2.96947, 15.963}}	{0.394889, 2.06929}	inf	3818.	3313.9	{1548., 6014.}	49.4449	0.228329	newly	1428.	1228.21	{565., 2281.}	49.9492	0.231985	Reff	0.209086	0.209106	{0.1926, 0.236192}	7.44724	ar	93873.	93829.	{93704., 93974.}	0.0964412
36	R0	{2.18718, 14.261}	{2.96398, 17.7348}	{{2.96398, 17.7348}, {2.96398, 17.7348}}	{35.5161, 24.3587}	inf	3033.	2182.05	{1015., 3916.}	52.2569	0.286354	newly	1295.	739.032	{351., 1319.}	57.4094	0.3641	Reff	0.381269	0.200378	{0.189547, 0.213495}	47.4444	ar	84379.	93780.3	{93638., 93879.}	11.1417
37	R0	{2.44912, 9.31118}	{2.93597, 11.6547}	{{2.93597, 11.6547}, {2.93597, 11.6547}}	{19.8786, 25.1689}	inf	559.	1227.9	{640., 2384.}	120.218	0.287326	newly	306.	586.148	{297., 1128.}	94.2194	0.236802	Reff	0.289559	0.197088	{0.190838, 0.204197}	31.9351	ar	88442.	93584.1	{93441., 93733.}	5.81405
38	R0	{2.05949, 5.72514}	{2.93597, 11.6547}	{{2.93597, 11.6547}, {2.93597, 11.6547}}	{42.5581, 103.571}	inf	81.	1227.9	{640., 2384.}	1415.93	1.12362	newly	76.	586.148	{297., 1128.}	671.247	0.829241	Reff	0.380141	0.197088	{0.190838, 0.204197}	48.1539	ar	81587.	93584.1	{93441., 93733.}	14.7046
39	R0	{1.75505, 8.52404}	{2.96978, 18.8857}	{{2.96978, 18.8857}, {2.96978, 18.8857}}	{69.2134, 121.558}	inf	1029.	6683.52	{3070., 11944.}	549.516	0.74046	newly	719.	2168.58	{1013., 3905.}	201.611	0.403846	Reff	0.527357	0.24369	{0.204974, 0.291395}	54.5395	ar	70925.	93825.4	{93652., 93970.}	32.2882
40	R0	{2.24724, 13.7861}	{2.8939, 16.8013}	{{2.8939, 16.8013}, {2.8939, 16.8013}}	{28.7757, 21.8713}	inf	2420.	3161.07	{1547., 5860.}	52.8648	0.165512	newly	1045.	1127.92	{565., 2167.}	47.3386	0.181591	Reff	0.360008	0.219346	{0.204483, 0.241959}	39.072	ar	85257.	93276.2	{93112., 93387.}	9.40595
41	R0	{1.77635, 8.62406}	{2.96947, 15.963}	{{2.96947, 15.963}, {2.96947, 15.963}}	{67.1669, 85.0984}	inf	882.	3313.9	{1548., 6014.}	275.726	0.497141	newly	608.	1228.21	{565., 2281.}	104.985	0.240382	Reff	0.494927	0.209106	{0.1926, 0.236192}	57.7501	ar	72941.	93829.	{93704., 93974.}	28.6368
42	R0	{1.80397, 5.4932}	{2.93597, 11.6547}	{{2.93597, 11.6547}, {2.93597, 11.6547}}	{62.7505, 112.166}	inf	102.	759.23	{393., 1488.}	644.343	0.814579	newly	82.	359.738	{187., 685.}	338.705	0.584909	Reff	0.48442	0.193639	{0.188401, 0.199147}	60.0266	ar	73232.	93584.1	{93441., 93733.}	27.7912
43	R0	{2.83096, 16.7077}	{2.93978, 19.9817}	{{2.93978, 19.9817}, {2.93978, 19.9817}}	{3.84393, 19.5958}	inf	3887.	7631.56	{3968., 13084.}	98.332	0.240405	newly	1377.	2371.75	{1221., 4091.}	78.2599	0.199322	Reff	0.237546	0.260923	{0.216603, 0.317114}	15.9695	ar	92740.	93627.3	{93451., 93834.}	0.956738
44	R0	{2.73678, 11.6021}	{2.9884, 12.8326}	{{2.9884, 12.8326}, {2.9884, 12.8326}}	{9.19402, 10.6058}	inf	851.	1014.65	{524., 1633.}	45.6844	0.152777	newly	393.	449.081	{230., 733.}	44.2871	0.154825	Reff	0.225237	0.186698	{0.182263, 0.191407}	17.1105	ar	92003.	93983.5	{93851., 94123.}	2.15266
45	R0	{2.14235, 6.98601}	{2.99939, 13.7076}	{{2.99939, 13.7076}, {2.99939, 13.7076}}	{40.0047, 96.215}	inf	172.	1806.74	{942., 2965.}	950.431	0.959998	newly	135.	754.726	{390., 1262.}	459.056	0.685404	Reff	0.36707	0.19148	{0.182963, 0.199999}	47.8357	ar	82974.	94036.2	{93909., 94200.}	13.3322
46	R0	{2.23409, 9.21289}	{2.99911, 15.6207}	{{2.99911, 15.6207}, {2.99911, 15.6207}}	{34.243, 69.5527}	inf	866.	5245.42	{2498., 9386.}	505.707	0.714317	newly	520.	1990.37	{932., 3609.}	282.764	0.512713	Reff	0.341659	0.221586	{0.194792, 0.254684}	35.9965	ar	85163.	94013.6	{93846., 94150.}	10.3926
47	R0	{2.12258, 8.15265}	{2.93597, 11.6547}	{{2.93597, 11.6547}, {2.93597, 11.6547}}	{38.3208, 42.956}	inf	433.	1227.9	{640., 2384.}	183.58	0.395618	newly	280.	586.148	{297., 1128.}	110.31	0.267354	Reff	0.375293	0.197088	{0.190838, 0.204197}	47.4842	ar	82690.	93584.1	{93441., 93733.}	13.1746
48	R0	{2.71771, 10.4636}	{2.80569, 11.6897}	{{2.80569, 11.6897}, {2.80569, 11.6897}}	{3.23728, 11.7178}	inf	924.	1913.5	{891., 2882.}	109.185	0.266884	newly	498.	913.2	{425., 1344.}	88.4873	0.227943	Reff	0.232147	0.223614	{0.214018, 0.232928}	5.50239	ar	91716.	92572.9	{92404., 92749.}	0.934279
49	R0	{2.44369, 6.66477}	{2.9751, 8.95585}	{{2.9751, 8.95585}, {2.9751, 8.95585}}	{21.7462, 34.376}	inf	49.	240.339	{121., 338.}	390.487	0.627174	newly	43.	137.629	{68., 204.}	220.068	0.439262	Reff	0.273693	0.183772	{0.180321, 0.187104}	32.8548	ar	88827.	93878.8	{93754., 94012.}	5.68721
50	R0	{2.77383, 17.9775}	{2.93978, 19.9817}	{{2.93978, 19.9817}, {2.93978, 19.9817}}	{5.9827, 11.1484}	inf	3843.	7631.56	{3968., 13084.}	100.453	0.244701	newly	1236.	2371.75	{1221., 4091.}	94.4732	0.229372	Reff	0.244319	0.260923	{0.216603, 0.317114}	16.0045	ar	92334.	93627.3	{93451., 93834.}	1.40065
